# ForceAtlas2, a Continuous Graph Layout Algorithm for Handy Network Visualization Designed for the Gephi Software

**DOI:** 10.1371/journal.pone.0098679

**Published:** 2014-06-10

**Authors:** Mathieu Jacomy, Tommaso Venturini, Sebastien Heymann, Mathieu Bastian

**Affiliations:** 1 Sciences Po, médialab, Paris, France; 2 Equipex DIME SHS, Paris, France; 3 Gephi Consortium, Paris, France; 4 LIP6 - CNRS - Universite Pierre et Marie Curie, Paris, France; Manchester University, United Kingdom

## Abstract

Gephi is a network visualization software used in various disciplines (social network analysis, biology, genomics…). One of its key features is the ability to display the spatialization process, aiming at transforming the network into a map, and ForceAtlas2 is its default layout algorithm. The latter is developed by the Gephi team as an all-around solution to Gephi users’ typical networks (scale-free, 10 to 10,000 nodes). We present here for the first time its functioning and settings. ForceAtlas2 is a force-directed layout close to other algorithms used for network spatialization. We do not claim a theoretical advance but an attempt to integrate different techniques such as the Barnes Hut simulation, degree-dependent repulsive force, and local and global adaptive temperatures. It is designed for the Gephi user experience (it is a continuous algorithm), and we explain which constraints it implies. The algorithm benefits from much feedback and is developed in order to provide many possibilities through its settings. We lay out its complete functioning for the users who need a precise understanding of its behaviour, from the formulas to graphic illustration of the result. We propose a benchmark for our compromise between performance and quality. We also explain why we integrated its various features and discuss our design choices.

## Introduction

This paper addresses two different audiences. To Gephi users, we offer a complete description of the ForceAtlas2 algorithm and its settings. To the researchers or engineers interested in the development of spatialization algorithms, we offer a discussion of our choices of features and implementation.

If developing an algorithm is “research” and implementing it is “engineering”, then a specificity of Gephi overall, is that it is based in engineering rather than in research. This is why it looks so different to a software like Pajek. This is also why ForceAtlas2 is more about usability than originality.

Our contribution to the mathematics of network spatialization is limited to the benchmark of a specific implementation of adaptive speed (step length selection). This paper focuses more on how classical techniques fit together in the perspective of a rich user experience - and which techniques do not.

It is necessary to explain quickly how the user feedback led us to the specific orientation of ForceAtlas2 (a continuous algorithm). In the next sections we will explore the different techniques gathered in this layout, with some formal terminology and many illustrations. We will discuss our implementation of step length selection with examples and a benchmark. And finally we will offer a short discussion about the general design of the algorithm.

In 2008 we started to develop Gephi [1], a software to visualize and manipulate networks, at the Maison des Sciences de l’Homme in Paris under the direction of Dana Diminescu [2]. Our goal was to provide some network analysis methods to social scientists, that would not require learning graph theory.

Three reference softwares inspired us: Pajek [3], GUESS [4] and TouchGraph. TouchGraph offered a manipulative interface that we highly appreciated, but it had serious performance issues and the layout was not adapted to scale-free networks of a hundred nodes or more (high visual cluttering). Pajek is very powerful but not adapted to dynamic exploration (it is designed as a computation software, where visualization is a bonus). GUESS was the most adapted to our needs, being user-centric and implementing state-of-the-art spatialization algorithms such as GEM [5].

We do not explore here the reasons why we created Gephi rather than just using GUESS, since it is a much larger discussion. However, an important point for this paper is that we wanted a *continuous* layout, that runs homogeneously and which can be displayed. Visualizing the “live” spatialization is a key feature of Gephi. It provides a very intuitive understanding of the layout process and its settings. It allows users to have a trial-error approach to the layout, that improves the learning curve of Gephi.

We developed ForceAtlas2 by combining existing techniques. We did it “wildly”: we did not start from a systematic review of academic papers, and we eventually redeveloped existing techniques. We implemented features when they were needed by users, and we tried to incorporate user-friendly settings in the design. (When we reworked all the settings, we created a “version 2” of “ForceAtlas” to avoid a too confusing change. Both versions are still available in Gephi even if the first version is obsolete.) We focused ForceAtlas2 on fluency and quality, because fluency is required by Gephi’s interactive user experience, and because researchers prefer quality over performance.

The fundamentals of the algorithm are not sophisticated. As long as it runs, the nodes repulse and the edges attract. This push for simplicity comes from a need for transparency. Social scientists cannot use black boxes, because any processing has to be evaluated in the perspective of the methodology. Our features change the forces or how they are simulated, but keep this model of continuous force directed layout: forces apply continuously as long as the layout is running. We give more details about our reasons at this end of this paper.

Developing a continuous algorithm prevented us from implementing many powerful techniques. We cite here some techniques that we intentionally avoided for focusing reasons. Simulated annealing [6] cannot be fully implemented, nor can any auto-stop feature (like Yifan Hu [7], also implemented in Gephi). Our layout stops exclusively at the user’s request. Phased strategies, used for example by OpenOrd [8], are by definition incompatible, even if in this case it allows OpenOrd to spatialize much larger networks. Graph coarsening [7], [9] cannot be implemented for the same reason. Finally, strategies where forces do not apply homogeneously do not necessary fit, because the motion of the network during the layout is not as fluid and it impacts the user experience. It is especially the case of the old Kamada Kawai [10] and more recently GEM [5].

We abandoned many techniques by keeping ForceAtlas2 continuous. But most of these are actually optimizations, and our performances are still compatible with the size of networks managed by Gephi (as we will see). We were able to implement qualitative features that impact the placement of the nodes, such as a degree-dependent repulsion force suggested by Noack [11], gravitation, and other features. We also implemented the Lin-Log forces proposed by Noack, a great inspiration for us, since his conception of layout quality corresponds to researchers’ needs (a visual interpretation of modularity).

## Anatomy of ForceAtlas2

ForceAtlas2 is a force directed layout: it simulates a physical system in order to spatialize a network. Nodes repulse each other like charged particles, while edges attract their nodes, like springs. These forces create a movement that converges to a balanced state. This final configuration is expected to help the interpretation of the data.

The force-directed drawing has the specificity of placing each node depending on the other nodes. This process depends only on the connections between nodes. Eventual attributes of nodes are never taken into account. This strategy has its drawbacks. The result varies depending on the initial state. The process can get stuck in a local minimum. It is not deterministic, and the coordinates of each point do not reflect any specific variable. The result cannot be read as a Cartesian projection. The position of a node cannot be interpreted on its own, it has to be compared to the others. Despite these issues, the technique has the advantage of allowing a visual interpretation of the structure. Its very essence is to turn structural proximities into visual proximities, facilitating the analysis and in particular the analysis of social networks. Noack [12] has shown that the proximities express communities. Noack relies on the very intuitive approach of Newman [13], [14]: actors have more relations inside their community than outside, communities are groups with denser relations. Newman proposes an unbiased measure of this type of collective proximity, called “modularity”. Noack [12] has shown that force-directed layouts optimize this measure: communities appear as groups of nodes. Force-directed layouts produce visual densities that denote structural densities. Other types of layouts allow a visual interpretation of the structure, like the deterministic layout “Hive Plots” [15], but they do not depict the modular aspect of the structure.

### Energy Model

Every force-directed algorithm relies on a certain formula for the attraction force and a certain formula for the repulsion force. The “spring-electric” layout [16] is a simulation inspired by real life. It uses the repulsion formula of electrically charged particles (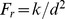) and the attraction formula of springs (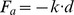) involving the geometric distance 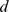 between two nodes. Fruchterman and Rheingold [17] created an efficient algorithm using custom forces (attraction 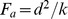 and repulsion 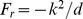, with 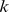 adjusting the scaling of the network). Note that actually, non-realistic forces have been used since the beginning, noticeably by Eades [16] in his pioneer algorithm. Fruchterman and Rheingold were inspired by Eades’ work, and they noticed that despite using the spring metaphor to explain his algorithm, the attraction force is not that of a spring.

Sixteen years later, Noack [11] explained that the most important difference among force-directed algorithms is the role played by distance in graph spatialization. In physical systems, forces depend on the distance between the interacting entities: closer entities attract less and repulse more than more distant entities and vice versa. The interdependence between distance and forces can be linear, exponential or logarithmic. The spring model for example, replicates precisely the physical forces from which it is inspired, thereby establishing a linear proportionality between the distance and the force (as for the spring attraction) and as a square proportionality between the distance and the force, as for electromagnetic repulsion. Noack defines the energy model or “(attraction,repulsion)-model” of a layout as the exponent taken by distance in the formulas used to calculate attraction and repulsion (the 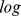 being considered as the 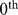 power). For example, the model of the spring-electric layout is 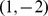.

**Figure 1:**
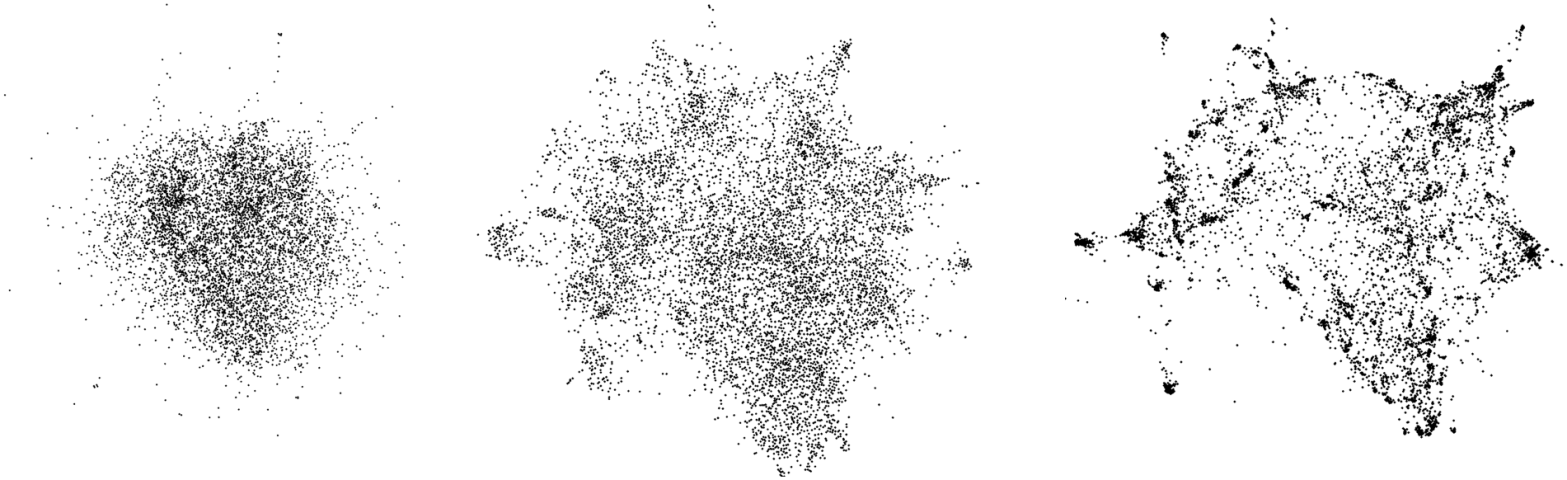
Layouts with different types of forces. Layouts with Fruchterman-Reingold (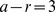), ForceAtlas2 (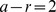) and the LinLog mode of ForceAtlas2 (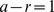).

The (attraction,repulsion)-model of ForceAtlas 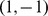 has an intermediate position between Noack’s LinLog 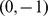 and the algorithm of Fruchterman and Rheingold 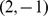, as pictured in Figure 1.

Noack [12] states that “distances are less dependent on densities for large 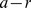, and less dependent on path lengths for small 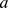
” (the “density” is the ratio of actual edges on potential edges). It means that visual clusters denote structural densities when 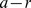 is low, that is when the attraction force depends less on distance, and when the repulsion force depends more on it. ForceAtlas2’s ability to show clusters is better than Fruchterman and Rheingold’s algorithm but not as good as the LinLog (Figure 1).

#### A classical attraction force

The attraction force 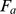 between two connected nodes 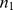 and 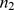 is nothing remarkable. It depends linearly on the distance 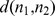. We will explain later why there is no constant adjusting of this force.
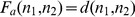
(1)


#### Repulsion by degree

A typical use case of ForceAtlas2 is the social network. A common feature of this type of network is the presence of many “leaves” (nodes that have only one neighbor). This is due to the power-law distribution of degrees that characterizes many real-world data. The forests of “leaves” surrounding the few highly connected nodes is one of the principal sources of visual cluttering. We take into account the degree of the nodes (the count of connected edges) in the repulsion, so that this specific visual cluttering is reduced.

**Figure 2:**
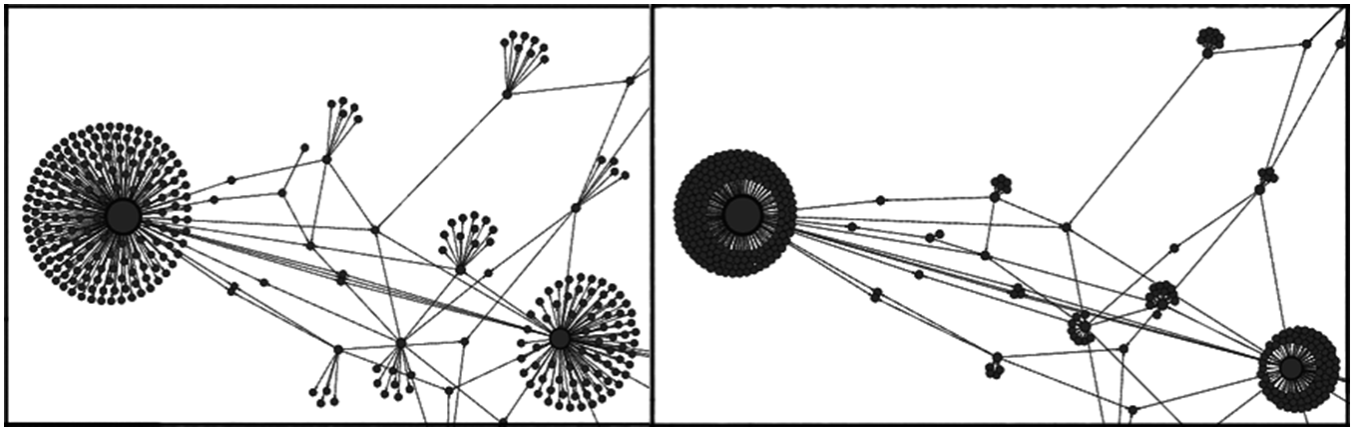
Regular repulsion vs. repulsion by degree. Fruchterman-Rheingold layout on the left (regular repulsion) and ForceAtlas2 on the right (repulsion by degree). While the global scheme remains, poorly connected nodes are closer to highly connected nodes. (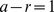).

The idea is to bring poorly connected nodes closer to very connected nodes. Our solution is to tweak the repulsion force so that it is weaker between a very connected node and a poorly connected one. As a consequence they will end up being closer in the balanced state (Figure 2). Our repulsion force 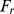 is proportional to the produce of the degrees plus one (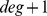) of the two nodes. The coefficient 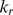 is defined by the settings.
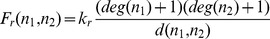
(2)


This formula is very similar to the edge repulsion proposed by Noack [11] except that he uses degree and not the degree plus one. The 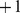 is important as it ensures that even nodes with a degree of zero still have some repulsion force. We speculate that this feature has more impact on the result and its readability than the (attraction, repulsion)-model.

### Settings

We detail now the settings proposed to the user, what they implement, and their impact on the layout. Most of these settings allow the user to affect the placement of nodes (the shape of the network). They allow the user to get a new perspective on the data and/or to solve a specific problem. They can be activated while the layout is running, thus allowing the user to see how they impact the spatialization.

#### LinLog mode

Andreas Noack produced an excellent work on placement quality measures [18]. His LinLog energy model arguably provides the most readable placements, since it results in a placement that corresponds to Newman’s modularity [14], a widely used measure of community structure. The LinLog mode just uses a logarithmic attraction force.
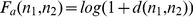
(3)


This formula is different from Noack’s since we add 1 to the distance to manage superposed nodes (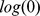 would produce an error). We have already seen that this energy model has a strong impact on the shape of the graph, making the clusters tighter (Figure 1). We also observed that it converges slowly in some cases. Switching from regular mode to LinLog mode needs a readjustment of the scaling parameter.

#### Gravity

**Figure 3:**
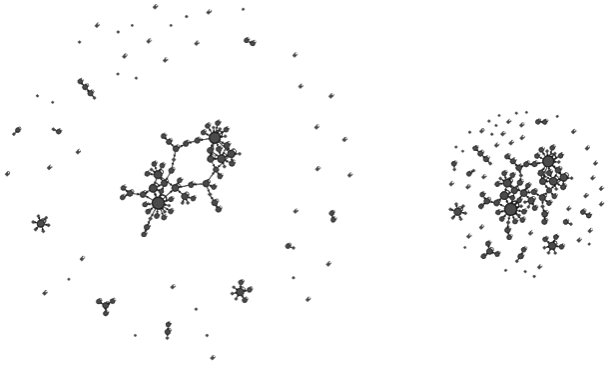
Effects of the gravity. ForceAtlas2 with gravity at 2 and 5. Gravity brings disconnected components closer to the center (and slightly affects the shape of the components as a side-effect).


Gravity is a common improvement of force-directed layouts. This force 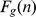 prevents disconnected components (islands) from drifting away, as pictured in Figure 3. It attracts nodes to the center of the spatialization space. Its main purpose is to compensate repulsion for nodes that are far away from the center. In our case it needs to be weighted like the repulsion:
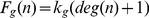
(4)


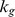
 is set by the user.

The “Strong gravity” option sets a force that attracts the nodes that are distant from the center more (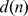 is this distance). This force has the drawback of being so strong that it is sometimes stronger than the other forces. It may result in a biased placement of the nodes. However, its advantage is to force a very compact layout, which may be useful for certain purposes.
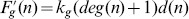
(5)


#### Scaling

**Figure 4:**
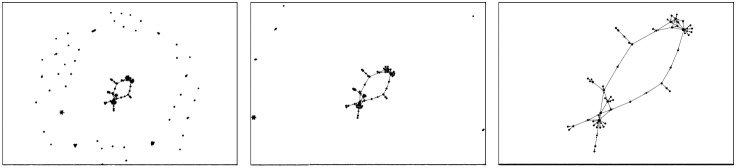
Effects of the scaling. ForceAtlas2 with scaling at 1, 2 and 10. The whole graph expands as scaling affects the distance between components as well as their size. Note that the size of the nodes remains the same; scaling is not zooming.

A force-directed layout may contain a couple of constants 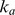 and 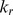 playing an opposite role in the spatialization of the graph. The attraction constant 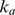 adjusts the attraction force, and 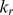 the repulsion force. Increasing 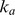 reduces the size of the graph while increasing 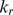 expands it. In the first version of ForceAtlas, the user could modify the value of both variables. For practical purposes, however, it is better to have only one single scaling parameter. In ForceAtlas2, the scaling is 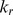 while there is no 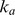. The higher 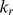, the larger the graph will be, as you can see in Figure 4.

#### Edge weight

**Figure 5:**
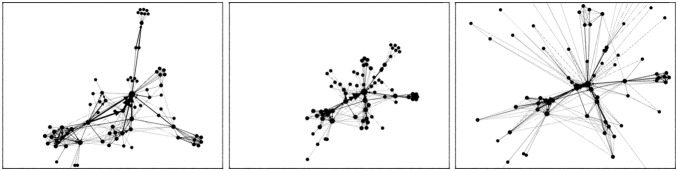
Effects of the edge weight influence. ForceAtlas2 with Edge Weight Influence at 0, 1 and 2 on a graph with weighted edges. It has a strong impact on the shape of the network.

If the edges are weighted, this weight will be taken into consideration in the computation of the attraction force. This can have a dramatic impact on the result, as pictured in Figure 5. If the setting “Edge Weight Influence” 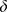 is set to 0, the weights are ignored. If it is set to 1, then the attraction is proportional to the weight. Values above 1 emphasize the weight effects. This parameter is used to modify the attraction force according to the weight 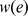 of the edge 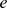:
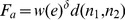
(6)


#### Dissuade Hubs

ForceAtlas2 has a “Dissuade Hubs” mode that, once activated, affects the shape of the graph by dividing the attraction force of each node by its degree plus 1 for nodes it points to. When active, the attraction force is computed as follows:
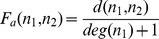
(7)


This mode is meant to grant authorities (nodes with a high indegree) a more central position than hubs (nodes with a high outdegree). This is useful for social networks and web networks, where authorities are sometimes considered more important than hubs. “Dissuade Hubs” tends to push hubs to the periphery while keeping authorities in the center. Note that here we improperly use the concepts of Hub and Authority defined by Kleinberg [19]. We do not actually compute the HITS algorithm for performance issues.

#### Prevent Overlapping

**Figure 6:**
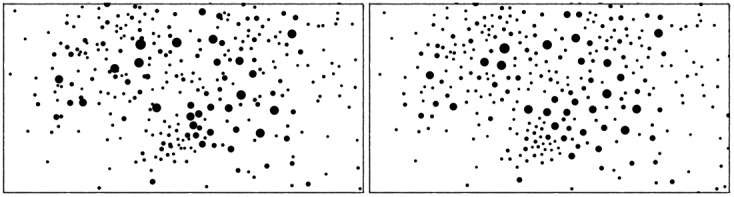
Effects of the overlapping prevention. ForceAtlas2 without and with the nodes overlapping prevention.

With this mode enabled, the repulsion is modified so that the nodes do not overlap. The goal is to produce a more readable and aesthetically pleasing image, as pictured in Figure 6.

The idea is to take into account the size of the nodes 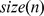 in computing the distance 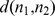 both in the attraction force and in the repulsion force.

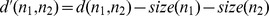
 is the “border-to-border” distance preventing overlapping.
- if 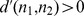 (no overlapping) then we use 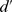 instead of 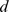 to compute forces:

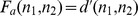


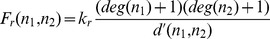

- if 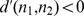 (overlapping) then no attraction and a stronger repulsion:

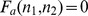


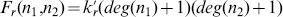

- if 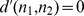 then there is no attraction and no repulsion

In Gephi’s implementation 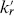 is arbitrarily set to 100. Note that the swinging measure is biased due to this option, that is why we also implemented a diminishing factor on the local speed (dividing it by 10). It is important to notice that this mode adds a considerable friction in the convergence movement, slowing spatialization performances. It is necessary to apply it only after the convergence of graph spatialization.

#### Approximate repulsion

In order to improve spatialization performances on big graphs, we implemented the optimization of Barnes Hut [20]. Relying on an approximate computation of repulsion forces, such optimization generates approximation and may be counter-productive on small networks, thus we allow the user to disable it. Besides from the side effects of the approximation, it does not impact the shape of the layout. Without the Barnes Hut optimization, the complexity time is 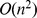 where 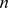 is the number of nodes.

## Performance Optimization

### The Issue of Speed

**Figure 7:**
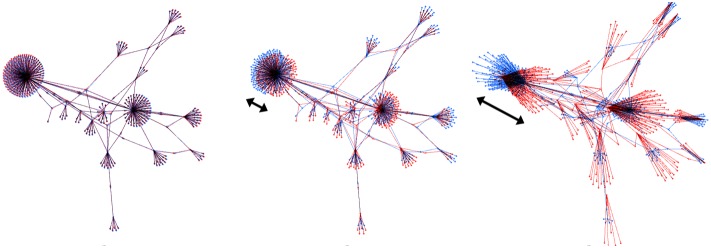
The oscillation of nodes increases with speed. Fruchterman-Rheingold layout at speeds 100, 500 and 2,500 (superposition at two successive steps).

When employing a force-based layout, users have to deal with a speed/precision trade-off. Speed may accelerate the convergence, but the lack of precision may prevent it. This issue is a consequence of using a simulation of the forces. It appears in any force-directed algorithm as well as in other types of simulations. The time is not something continuous in the simulation, because it is computed step-by-step. When using many computing steps, a precise simulation is produced but it takes longer to compute: it is slow. If few steps are chosen, it is computed quickly but the simulation is imprecise. Reducing the number of steps is making a rougher approximation of the system. The proper term to discuss this would be “step length”, since it is the mathematical variable that we actually use. But we will prefer here the term of “speed”, because it is closer to the experience of users. The speed of the simulation is just like the step length: a high speed means long steps (less precision), a low speed means short steps (more precision). In a force-directed algorithm, increasing the speed makes the precision drop. We cannot have speed and precision at the same time. The effect of the approximation is that some nodes become unable to find a stable position and start oscillating around their balancing position (Figure 7).

This oscillation problem is known as a problem of “temperature”, because we can compare the movement of a node to the temperature of a molecule. Different solutions exist: local temperatures as featured in GEM [5], adaptive cooling as featured in Yifan Hu [7] or simulated annealing [6]. ForceAtlas2 features its own implementation of local temperatures as well as adaptive cooling, but in the perspective of a continuous layout. In terms of “speed vs. precision”, since users are more comfortable with these concepts, we compute an optimal speed for each node as well as for the whole graph. Our strategy is to measure oscillations and to compute a speed that allows only a certain amount of oscillation. This amount is set by the user as “Tolerance (speed)”. In Gephi’s implementation, we set three default values: 0.1 under 5000 nodes, 1 up to 50000 nodes and 10 above 50000. We now describe how this feature works.

### Adapting the Local Speed

We implemented a strategy aimed at optimizing the convergence. Researchers often visualize scale-free networks where some nodes gather a huge amount of edges. Highly connected nodes have a high temperature. They tend to oscillate quickly, and require a high level of precision, thus a low speed. Poorly connected nodes are very stable and so can operate at high speed. If we have different speeds for different nodes, we can achieve a much better performance. Our strategy is to determine the speed of each node by observing its oscillation, like in GEM [5]. But our implementation is actually quite different.

Our version of oscillation is based on the forces applied to each node, and we call it “swinging” (oscillation is about distances). We define the swinging 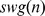 of a node 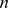 as the divergence between the force applied to 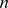 at a given step and the force applied 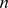 at the previous step. Intuitively, the more the node is asked to change direction, the more it swings. 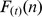 it the result force applied to 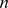 at step 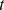.
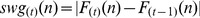
(8)


For a node moving towards its balancing position, 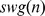 remains close to zero. A node that is diverging, on the other hand, has a high swinging and its movement needs to be slowed down to make it converge. The speed 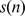 of a node 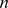 determines how much displacement 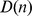 will be caused by the resultant force *F*(*n*): 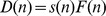. The resultant force is the sum of all forces applied to each node (attraction, repulsion and gravity: 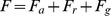). So in ForceAtlas2 the speed is different for every node, and computed as follows:
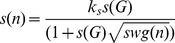
(9)



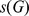
 is the global speed of the graph (see below). 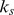 is a constant set to 0.1 in Gephi’s implementation.

The more a node swings, the more it is slowed. If there is no swinging, the node moves at the global speed. As a protection, we implemented an additional constraint that prevents the local speed from being too high, even in case of very high global speeds.
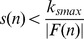
(10)



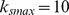
 in Gephi’s implementation.

### Adapting the Global Speed

At each step, two global values are computed and used to set the global speed: the global swinging and the global effective traction.

The global swinging 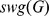 represents the quantity of erratic movement present in the global movement of the graph. It is the sum of local swinging values, weighted by the degree of each node as in our repulsion force (degree+1).
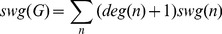
(11)


The effective traction 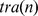 of a node is the amount of “useful” force applied to that node. Effective traction is the opposite of swinging: forces that contribute to the convergence. It is defined as an average:
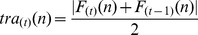
(12)


If a node keeps its course, then 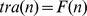. If it goes back to its previous position (a perfect swinging) then 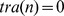.

The global effective traction 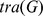 is the weighted sum of effective tractions of nodes:
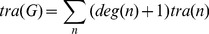
(13)


The global speed 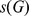 keeps the global swinging 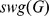 under a certain ratio 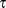 of the global effective traction 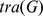 and is defined as follows:
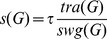
(14)


The ratio 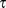 represents the tolerance to swinging and is set by the user.

NB: During our tests we observed that an excessive rise of the global speed could have a negative impact. That is why we limited the increase of global speed 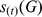 to 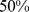 of the previous step 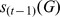.

### Details on this Strategy

Our initial idea was to get the optimal speed under every circumstance, and avoid a “speed” setting that users do not manage easily. We did not succeed, and we still have it under the name of “Tolerance”. Below, we explain the strategy we adopted. An optimal global speed is a similar idea to simulated annealing [6]. However, it is not the same because we have to prevent the freezing of the network. Simulated annealing is to find the right way to “cool down” the network, to reduce its speed so that it convergences more efficiently. Intuitively, the network can go faster at the beginning, since it is about finding its general shape, and needs more precision in the end, for the details. In more scientific terms, simulated annealing is about shortening the steps in the end for refining the spatialization, and stopping it. Yifan Hu [7] uses this technique at the end, during a refining phase. However he remarks that “for an application of a force-directed algorithm from a random initial layout, an adaptive step length update scheme is more successful in escaping from local minimums. […] Step length can increase as well as reduce, depending on the progress made”. Yifan Hu remarks that out of the refining phase, an adaptive speed is about “heating” as well as “cooling”, because escaping a local minimum may need more speed (heating). This applies to our scenario, since there is no refining phase in a continuous algorithm. Yifan Hu evaluates the convergence of the network and adapts its speed in consequence. Our “global speed” plays the same role, but we evaluate the convergence differently. Yifan Hu relies of the variation of global energy, while we rely on the regularity of applied forces (effective traction). The reason is that we have also a local speed optimization, like GEM [5], and that we need some homogeneity between the global speed and the local speed.

We explained that the local speed aims at providing more precision to nodes that fail at converging. Like GEM, we try to minimize swinging (oscillations). The local speed can slow the nodes down, but cannot speed them up. Even if the node requires more speed, it is limited by the global speed. The global speed determines the global movement, it is an “adaptive heating” rather than an “adaptive cooling”. It is as high as possible, in the limit of a certain amount of global swinging determined by the “Tolerance” setting. The local speed regulates the swinging while the global speed regulates the convergence. But the regulation of convergence is indirect, since we just compare the global effective traction with the swinging. We rely here on the assumption that oscillation denotes a lack of convergence. This assumption is reasonable, even if we know that it is false under certain circumstances (the swinging of a node propagates to its neighbors). GEM also relies on this assumption.

### Comparison with other Algorithms

Here, we compare ForceAtlas2 to the recent algorithm of Yifan Hu and to the old and classic layout of Fruchterman and Reingold. We did not compare it to OpenOrd, which is very efficient, but is not a continuous layout. Nor did we compare it to GEM because it is not implemented in Gephi (that we used as a benchmarking tool). We also compared the LinLog variant of ForceAtlas2 because we had no other implementation (they are very close).

We want to evaluate the speed as well as the quality of each algorithm on different networks. Different measures exist to evaluate the quality of a spatial arrangement of the nodes. H. Purchase uses aesthetic criteria [21] while A. Noack prefers interpretive properties [18]. We chose Noack’s “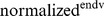 atedge length” (15) because it is more adapted to scale-free networks and has been used by Noack to evaluate Fruchterman-Reingold and LinLog.
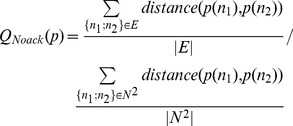
(15)


We will observe that contrary to our expectations, Fruchterman-Reingold performs better than LinLog, while LinLog is empirically more readable than Fruchterman-Reingold (we provide more details below). However this m easure is very good at capturing the process of a layout algorithm applied to a given network. Unlike other measures like edge crossings [21], it is sensitive to the smallest displacements. We rely on it to track the behavior of each benchmarked algorithm and to identify when the convergence is reached. Even if the measure is not fully satisfying to evaluate the quality, it is a good way to evaluate the speed.

Noack’s measure has another drawback. It is better when it is lower, which may lead to interpretation issues. We decided to invert the measure to be clearer (16):
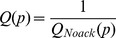
(16)


**Figure 8:**
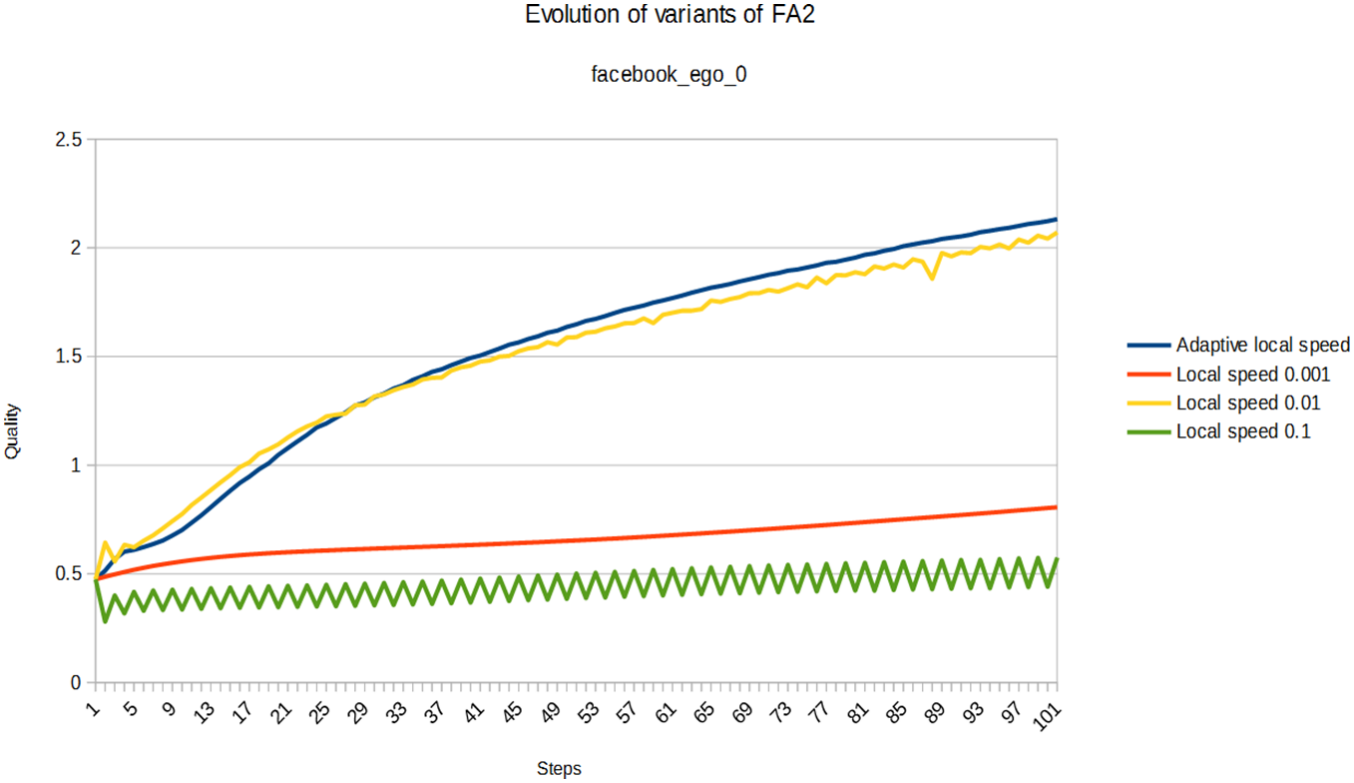
Adaptive local speed is a good compromise. Evolution of the quality of ForceAtlas2 variants at each iteration (the higher the better). Different values of the local speed give different behaviors. The adaptive local speed achieves the best compromise between performance and quality. The network used is “facebook_ego_0” from our dataset.

**Figure 9:**
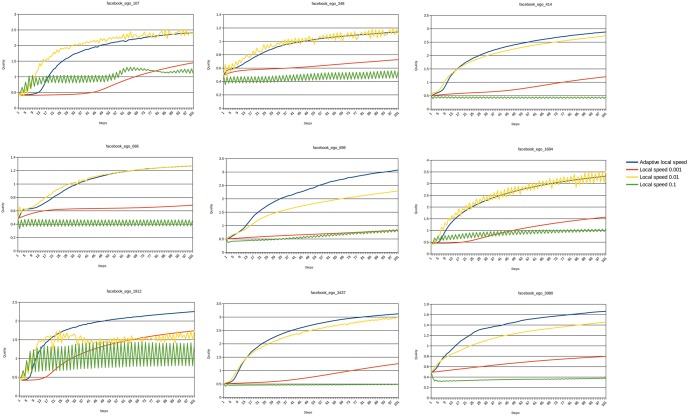
Effects of adaptive local speed on different networks. Evolution of the quality of ForceAtlas2 variants at each iteration on the other facebook ego-networks of our dataset. The adaptive local speed is always the best. Local speed 0.001 converges poorly because the speed is too low. Local speed 0.1 converges poorly because it oscillates a lot: the speed is too high. Local speed 0.01 is sometimes adapted to the network, and sometimes not, but never outperforms the adaptive speed.

At each step of the tested algorithm, we compute the quality for current positions. All the layouts, by definition, improve the quality of the spatialization. We compare the best quality they reach, and how many steps are needed to reach a good convergence (performance). Figure 8 pictures the impact of the adaptive local speed feature using this protocol. The featured network is “facebook_ego_0” from our dataset. We compare the actual implementation to variants where we fixed the local speed at different values (the algorithm is otherwise similar to the implementation described above). We observe different scenarios. If the speed is too low (0.001), the convergence is slow and we do not have enough steps to see the final quality. If the speed is too high (0.1) the quality stagnates early on, because of oscillations. A medium value of 0.01 has a good convergence and a good final quality, but the adaptive local speed achieves even better on convergence as well as on final quality. We reproduced this protocol on the other facebook ego-networks of the dataset and the results confirm this behavior, as pictured in Figure 9.

We benchmarked our algorithm with a dataset of 68 networks from 5 to 23,133 nodes. We tried to gather varied networks corresponding to the actual use of Gephi (a lot of social networks, and scale-free networks in general). Most of these networks are from the Stanford Large Network Dataset Collection (http://snap.stanford.edu/data/) and include social networks (Facebook and Twitter ego-networks), collaboration networks (from Arxiv) and autonomous systems (peering information inferred from Oregon route-views). Some networks come from the Gephi datasets, and include biological networks (neural network of C. Elegans, protein-protein interaction network in yeast). The others are generated with Gephi and include trees, random networks and small-world networks. Our dataset and the description of each network are included in the online repository of the benchmark (https://github.com/medialab/benchmarkForceAtlas2).

We compared four different algorithms: ForceAtlas2 (FA2), its LinLog variant (FA2_LL), Fruchterman-Reingold (FR) and Yifan Hu (YH). We used the default settings, with the exception of a few settings. FA2 and FA2_LL have different settings for small, medium and large networks: we used the medium settings on every network. FR was so slow that we updated its speed to 10 and its “Area” setting to 1000 so that the resulting layout has a comparable size. We also set the FA2_LL “Scaling” to 0.1 for the same reason.

Exact settings are:

- FA2: BarnesHutTheta 1.2; EdgeWeightInfluence 1.0; Gravity 0.0; JitterTolerance 1.0; ScalingRatio 2.0; AdjustSizes false; BarnesHutOptimize true; LinLogMode false; OutboundAttractionDistribution false; StrongGravityMode false
- FA2_LL: BarnesHutTheta 1.2; EdgeWeightInfluence 1.0; Gravity 0.0; JitterTolerance 1.0; ScalingRatio 2.0; AdjustSizes false; BarnesHutOptimize true; LinLogMode true; OutboundAttractionDistribution false; StrongGravityMode false
- YH: BarnesHutTheta 1.2; ConvergenceThreshold 1.0E-4; InitialStep 20.797; OptimalDistance 103.985; QuadTreeMaxLevel 10; RelativeStrength 0.2; StepRatio 0.95; AdaptiveCooling true
- FR: Area 1000.0; Gravity 0.0; Speed 10.0

Even if some of the networks are large (23,133 nodes and 186,936 edges) while others are very small (5 nodes and 5 edges), we wanted to use the same benchmark protocol. On the one hand, computing the layout quality is time-consuming on the biggest networks, and the convergence is slow (more than 1,000 steps). We did not have the time to compute the layout quality for hundreds of steps on each network. On the other hand, the small networks converge in a few steps, and we wanted to be able to spot the moment it happens. We had to track the early steps. As a compromise, we decided to compute layout quality each “power of 2” step: 1, 2, 4, 8… up to 2048. The quality evolves a lot at the early stages of the spatialization, and then reaches a more static state. Our protocol provides the early behavior of each algorithm as well as its long-term results. We also observed that some layouts cause oscillations (as pictured in Figure 8), so we computed each “power of 2 plus one” step: 2, 3, 5, 9… up to 2049. We averaged the quality at each “power of 2” step with the next step to remove oscillations.

**Figure 10:**
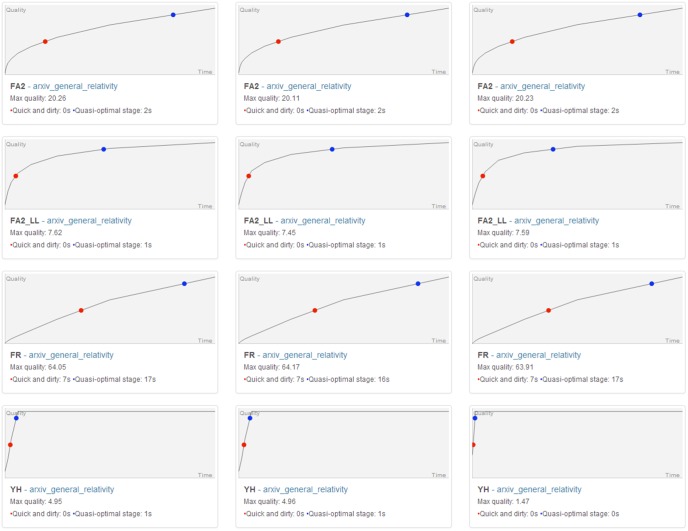
Records for a single network. Evolution of the layout quality for a single network over 2048 steps. Rows are the 4 different layouts and columns the 3 different randomizations. The red dot is the “Quick and dirty point” where 50% of the maximum quality is reached, and the blue dot is the “Quasi-optimal point” where 90% of the maximum quality is reached. The full visualization is available at this URL: https://github.com/medialab/benchmarkForceAtlas2/tree/master/benchmarkResults.

Each network was randomized three times. The three random assignments are saved in the dataset. You can download the dataset here: (https://github.com/medialab/benchmarkForceAtlas2/blob/master/dataset.zip). The benchmark resulted in 816 records of the layout quality at different steps. You can visualize these records there http://medialab.github.io/benchmarkForceAtlas2/ and download them https://github.com/medialab/benchmarkForceAtlas2/tree/master/benchmarkResults. Each file was analyzed to find the maximum quality, and two key moments. The “Quick and dirty” point is reached at 50% of the maximum quality, while the “Quasi-optimal” point is reached at 90% of the maximum quality. The first corresponds to an estimation of a rough spatialization while the second approximates a satisfying layout. A sample of these records is pictured in Figure 10, and the full visualization is available online. We expressed these points in milliseconds using the timestamps in the records. The maximum quality, the “Quick and dirty” time (QND Time) and the “Quasi-optimal” time (QO Time) are averaged over the 3 randomizations for each layout.

**Figure 11:**
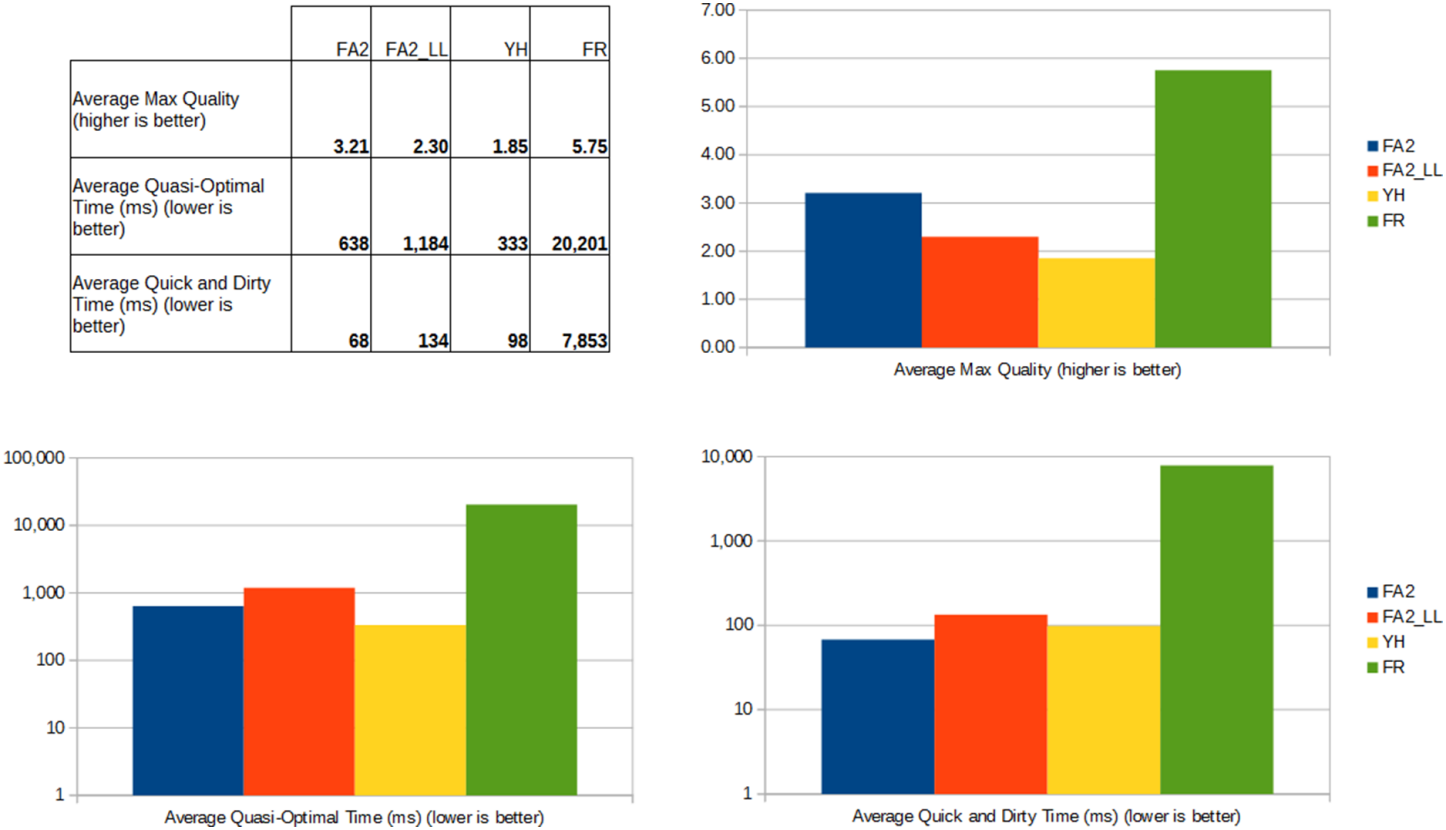
Overall results of the benchmark. Note that the second and third charts have logarithmic scales. FR is really slow, YH has a good performance and FA2 has a good quality.

**Figure 12:**
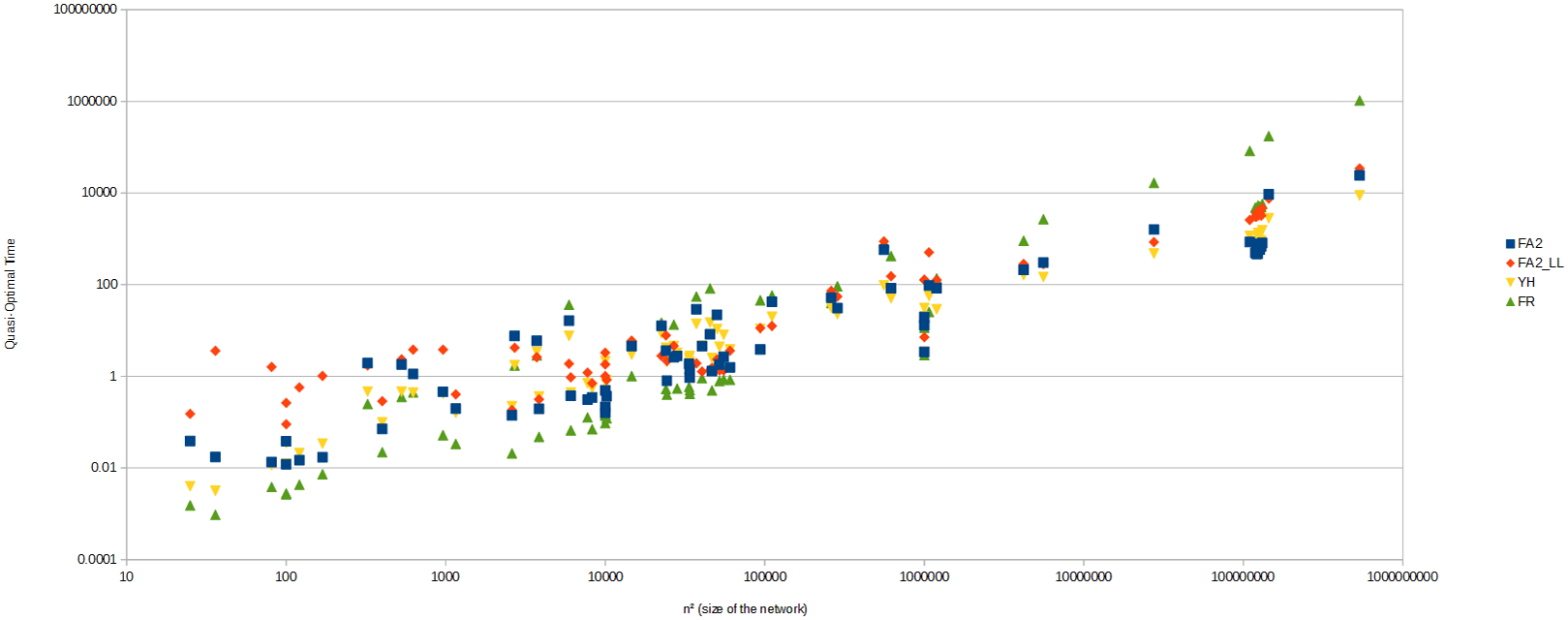
Quasi-Optimal Time over network size. The lower is the better. Note that both scales are logarithmic. On small networks, FR is the best while FA2_LL is slower. On large networks, FR has a poor performance while other algorithms perform similarly on large networks.

The overall results, as pictured in Figure 11, show that FR reaches the best quality but is too slow. Its performance is so poor on large networks that it cannot be used without an optimized implementation. YH, FA2 and FA2_LL have a comparable quality and performance, and Yifan Hu is quicker while ForceAtlas2 has a better quality. The details give us some useful informations about the specificities of each algorithm. Yifan Hu has the best performance, with an average QO Time of 333 ms, followed by ForceAtlas2 (638 ms), the LinLog variant (1,184 ms) and finally Fruchterman-Reingold (20,201 ms). FR is not optimized and was really slow on the largest networks. The Figure 12 shows the differences of algorithms depending on the size of the network. FR is most suitable for smaller networks and the worst for the largest. FA2 and YH are similar at all scales while FA2_LL is significantly worse on small networks, but not so much on the largest. FA2 is the quickest to reach its Quick and Dirty point in average (68 ms). YH (98 ms) and FA2_LL (134 ms) are not much different, but they highlight the good convergence of FA2 in the early steps. FR is also far behind (7,853 ms).

**Figure 13:**
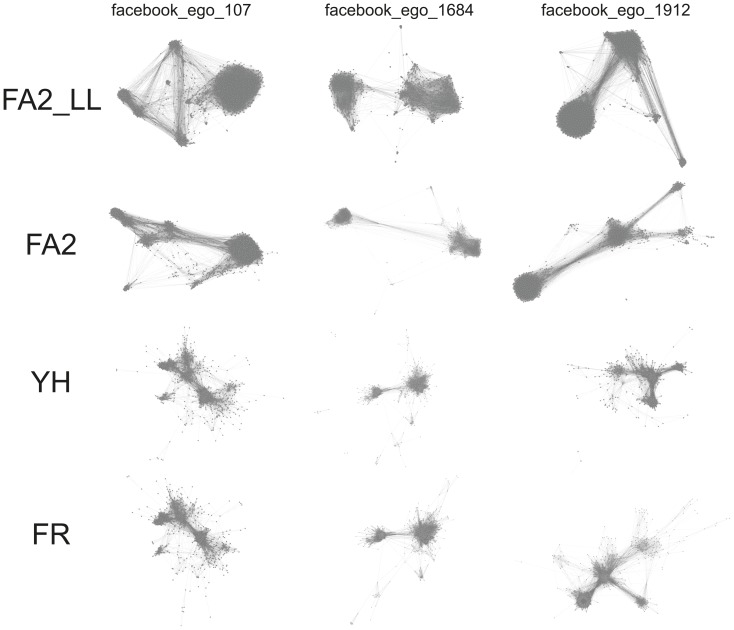
Layouts give visibly different results. We find that FA2_LL and FA2 are more readable, because the different areas of the network are more precisely defined. However, we do not know any quality measure that captures this phenomenon.

We find empirically that it is easier to identify the clusters in FA2 and FA_LL than in FR and YH. Noack’s measure does not reflect this observation, and we do not know how to measure this phenomenon. However we think it is useful to show a sample result of each layout. The Figure 13 compares the result of the four layouts in three different cases (Facebook ego-networks). We find that the different areas of the network are more precisely defined with FA2_LL and FA2. Even if this is debatable, it is clear that the layouts have different visual properties that are not captured by the quality measure we used. A more advanced benchmark would require a different way to capture the visual properties of the layouts.

In conclusion ForceAtlas2 compares to Yifan Hu in terms of quality and performance. Yifan Hu has a better performance on small networks while ForceAtlas2 has a better measured quality, though evaluating the readability of different layouts would require a different discussion and protocol. The LinLog mode of ForceAtlas2 brings more quality at the price of performance, and Fruchterman-Reingold performs poorly on large networks.

## Discussion: Designing a Generic, Continuous Layout

The visualization of a network involves design choices. We think users have to be aware of the consequences of these choices. The strategy we adopt in Gephi is to allow users to see in real time the consequences of their choices, learning by trial and error. Interaction, we believe, is the key to understanding. While developing the Gephi user experience, we strongly desired a “live” spatialization process. Hiding it may lead users to believe that the placement is unique or optimal. Non-expert users need to observe the spatialization process and even to interact with it. Manipulating a graph while it spatializes helps to understand the difference between a graph layout and a Cartesian projection. The effect of the settings can be observed and understood. It helps to figure out that spatialization is a construction that involves the responsibility of the user.

Users can act on the network by changing the ranking of the nodes, or filtering nodes and edges, even creating new entities. ForceAtlas2 passes on modifications in real time, re-computing forces and continuously updating the placement of nodes. It is possible to “play” with the network. Since it is intuitive for users, developers can integrate other features on top. For instance we integrate the visualization of a dynamic network just as a particular case of dynamic filtering: the real-time layout updates the structure according to the specified time span. For an example see the dynamic visualization of a Twitter conversation, http://gephi.org/2011/the-egyptian-revolution-on-twitter.

Data monitoring is a basic use case of network visualization. With Gephi we intend to foster advanced uses: data exploration and map making. These uses are more demanding. Exploring the data may require searching for an adapted layout: a satisfactory algorithm with satisfactory settings. We cannot discuss here how and why some algorithms are better choices for certain networks, but we can give basic example cases. ForceAtlas2 is not adapted to networks bigger than 100,000 nodes, unless allowed to work over several hours. On the contrary, OpenOrd [8] is not adapted to networks of fewer than 100 nodes, because its side effects are too visible at this scale. Certain algorithms are more adapted to certain sizes, as well as certain densities, or certain community structure. Certain energy models provide a better depiction of certain network types. Alternative energy models are relevant features to diversify the algorithm’s applications. The *LinLog*, *edge weight* and *gravity* settings are such options, fostering a better exploration of the structure. Map making requires different features. Its purpose is to make the network fit in a limited graphic space. *Scaling* and *gravity* settings help users to produce a more compact network. The *overlapping prevention* provides more readability to the result. Finally, some features are implemented just for performance, such as the Barnes Hut’s optimization (*approximate repulsion*) and adaptive speeds. Even in this case we try to provide explicit settings to the user (*Tolerance (speed)*).

Integrating various features forces us to adapt some of them. We bring homogeneity in the different forces we implement. First we weight the nodes by degree plus one instead of just the degree (we cannot ignore nodes of degree 0). Secondly we adapt the *gravity* energy model to the repulsion force to limit its side effects. When repulsion is weighted in a certain way (for instance with the *dissuade hubs* setting) then the gravity is weighted the same way. We also normalized certain features to provide a smoother user experience. When *dissuade hubs* is activated, we compute a normalization to ensure that the total energy with the alternative forces is the same to the reference forces. Thanks to this trick, the network keeps a comparable spreading in the graphic space. Not that the *LinLog* energy model does not benefit from such a normalization, so you have to adjust the scaling when you activate it.

## Conclusion

As more and more people deal with relational data, network visualization assumes a key importance. ForceAtlas2 is our practical contribution to network sciences. It is not based on a new conception of force-directed layouts but it implements many features from other well-known layouts [7] [11] [5]. However, by its design and features, it aims to provide a generic and intuitive way to spatialize networks. Its implementation of adaptive local and global speeds gives good performances for network of fewer than 100000 nodes, while keeping it a *continuous* layout (no phases, no auto-stop), fitting to Gephi user experience. Its code is published in Java as a part of Gephi source code (https://github.com/gephi/gephi/tree/master/LayoutPlugin/src/org/gephi/layout/plugin/forceAtlas2).
